# Oxygen-Containing Functional Groups Regulating the Carbon/Electrolyte Interfacial Properties Toward Enhanced K^+^ Storage

**DOI:** 10.1007/s40820-021-00722-3

**Published:** 2021-09-13

**Authors:** Yufan Peng, Zhen Chen, Rui Zhang, Wang Zhou, Peng Gao, Jianfang Wu, Hui Liu, Jilei Liu, Aiping Hu, Xiaohua Chen

**Affiliations:** 1 College of Materials Science and Engineering, Hunan Joint International Laboratory of Advanced Materials and Technology of Clean Energy, Hunan Province Key Laboratory for Advanced Carbon Materials and Applied Technology, Hunan University, Changsha, 410082 People’s Republic of China; 2 Electrochemistry I, Helmholtz Institute Ulm (HIU), 89081 Ulm, Germany; 3 Karlsruhe Institute of Technology (KIT), P.O. Box 3640, 76021 Karlsruhe, Germany; 4 College of Chemistry and Material Science, Hunan Agricultural University, Changsha, 410128 People’s Republic of China

**Keywords:** Oxygen-containing functional groups, Solid electrolyte interphase, In situ spectroscopic characterization, Potassium ion batteries

## Abstract

Oxygen-containing functional groups were found to effectively boost the K^+^ storage performance of carbonaceous materials, however, the mechanism behind the performance enhancement remains unclear. Herein, we report higher rate capability and better long-term cycle performance employing oxygen-doped graphite oxide (GO) as the anode material for potassium ion batteries (PIBs), compared to the raw graphite. The in situ Raman spectroscopy elucidates the adsorption-intercalation hybrid K^+^ storage mechanism, assigning the capacity enhancement to be mainly correlated with reversible K^+^ adsorption/desorption at the newly introduced oxygen sites. It is unraveled that the C=O and COOH rather than C-O-C and OH groups contribute to the capacity enhancement. Based on in situ Fourier transform infrared (FT-IR) spectra and in situ electrochemical impedance spectroscopy (EIS), it is found that the oxygen-containing functional groups regulate the components of solid electrolyte interphase (SEI), leading to the formation of highly conductive, intact and robust SEI. Through the systematic investigations, we hereby uncover the K^+^ storage mechanism of GO-based PIB, and establish a clear relationship between the types/contents of oxygen functional groups and the regulated composition of SEI.

**Supplementary Information:**

The online version contains supplementary material available at 10.1007/s40820-021-00722-3.

## Highlights

- Oxygen functional groups improve rate capability as well as long-term cycling stability of graphite oxide.
- The adsorption-intercalation hybrid K^+^ storage mechanism of graphite oxide (GO) is elucidated by in situ Raman spectroscopy and systematic electrochemical characterization.
- It is unraveled that the C = O and COOH rather than C-O-C and OH groups contribute to the formation of highly conductive, intact and robust solid electrolyte interphase.

**Supplementary Information:**

The online version contains supplementary material available at 10.1007/s40820-021-00722-3.

## Graphical abstract

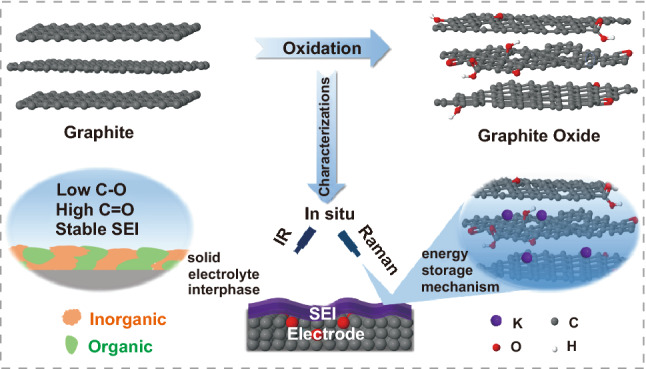

## Introduction

Potassium ion batteries (PIBs) have received increasing attention as an alternative to lithium ion batteries (LIBs) due to abundant reserve of potassium (> 1200 times more than Li in the Earth’s crust), decent redox potential of K^+^/K (E^0^_K_^+^_/K_ = -2.93 V vs. E^0^_Li_^+^_/Li_ = -3.04 V), and smaller Stokes' radius of K^+^ (3.6 Å compared to that of Li^+^ (4.8 Å)). These properties endow K^+^ storage devices with the advantages of low cost, moderately high working voltage, as well as fast K^+^ transport kinetics. However, since K^+^ has a larger ionic radius, 1.38 vs 0.76 Å (Li^+^), the development of K-based host materials with large interlayer distance is needed. Enormous investigations have been dedicated to, for example, carbonaceous materials because they are cheap, electrically conductive and highly controllable in terms of structure/composition [1, 2]. Early in 2015, highly reversible K^+^ de-/intercalation in graphite was reported [3–5]. However, it suffers from poor cycle life and unsatisfied rate capability toward K^+^ storage due to its long-range ordered structure and limited layer spacing.

It has been widely demonstrated that doping heteroatoms (such as N, S or P) are an effective approach to regulate physical and chemical properties, including bonding schemes, electronic structure, defects or ion adsorption ability, and thus improve the electrochemical properties of carbonaceous materials [6–8]. This strategy has been proven to be effective in energy storage applications (e.g., lithium/sodium-ion batteries, supercapacitors and oxygen reduction/evolution reaction (ORR/OER)) [9–12]. In the aspect of K^+^ energy storage systems, heteroatoms-doped carbon fibers [1], graphene [13] or hard carbon [14] exhibit excellent cyclability and rate performance. N-doped porous carbon nanosheets [15] show ultra-stable K^+^ storage capability because its abundant edge-N facilitates the surface-driven pseudocapacitive behavior alleviating structural degradation.

There is no doubt that the oxygen doping also plays a significant role in tailoring the properties of carbonaceous materials for diverse energy storage applications [16, 17]. The plasma-etched carbon cloth (P-CC) with defects and oxygen doping show enhanced catalytic activities for oxygen electrocatalysis [18]. Theoretically, it is revealed that the defective graphene containing carboxyl (COOH) species have the lowest ORR and OER overpotential. As the cathode for sodium-ion capacitors, the oxygen functional groups in densified graphene electrode are the dominant source of capacitive charge storage. The anodic redox peak between 2 ~ 3 V is attributed to the reversible redox reaction between sodium-ion and carbon–oxygen double bond (O-C = O/C = O) [17]. Similar behavior also occurs in LIBs. This Faradaic reaction with an equilibrium voltage of 3.2 V vs. Li can be attributed to reactions between Li^+^ and carbonyl, ester and carboxylic groups such as Li^+^  + C = O + e^−^ ⇋ C-O-Li [19]. The driving mechanism of the O-doping lies with the introduction of various functional groups and structural defects into carbon materials’ skeleton, which might serve as active sites for energy storage and facilitate the ion transport/diffusion. Interestingly, oxygen functional groups raise the carbon electrode impedance, but significantly boost the energy storage capability of the carbon electrode, indicating that decreasing electrode conductivity does not compromise the effectiveness of oxygen functional groups in energy storage [20, 21]. The in-depth influence of the oxygen functional groups on the impedance is worthy of further study.

By far, little attention has been paid to applying oxygen doping for K^+^ storage devices. Nanjundan et al. reported that oxygen-containing functional groups are favorable binding sites for K^+^ to boost the potassium ion storage performance [22]. Among different oxygen configurations, e.g., epoxide (C–O–C) and hydroxyl (OH) locate on both basal planes and edges of an aromatic domain, while carbonyl (C = O) and COOH decorate at the edges of an aromatic domain. The types/contents of oxygen-containing functional groups can affect the physicochemical properties (defects, edges, graphitic structure, conductivity, etc.) of electrode materials, and thus play an important role in regulating the electrode/electrolyte interface and electrochemical performance [23, 24]. Unfortunately, the fundamental mechanism of different oxygen functional groups on the electrochemical performance enhancement and solid electrolyte interphase (SEI) formation remain unclear.

Herein, comprehensive characterizations including in situ Fourier-transform infrared (FT-IR) spectra, together with in situ electrochemical impedance spectroscopy (EIS) were employed to correlate the oxygen functional group types/contents with K^+^ storage in GO. These functional groups remarkably expand the interlayer spacing of bulk graphite and tailor the physicochemical properties. Additionally, the oxygen-containing functional groups are found to be active for K^+^ adsorption in the potential range of 3.0–0.7 V, and facilitate the formations of SEI layers that are high ionic conductive, intact and stable. All these, contribute to the improved rate performance and cycling stability. Moreover, the K^+^ storage behavior is strongly dependent on both the types of oxygen-containing functional groups, i.e., the COOH and C = O functional groups, as well as their contents. Systematic studies have shown that high C-O content significantly promotes the formation of SEI organic component (ROCO_2_K) and is negatively correlated with potassium storage performance, while C = O and COOH promote the generation of SEI inorganic component (K_2_CO_3_) and are beneficial to potassium storage performance. In addition, the K^+^ storage mechanisms of graphite oxide (GO) are also identified from in situ Raman characterizations. Our results establish a clear relationship between the types/contents of oxygen functional groups and K storage performance, promotes better fundamental understanding of K^+^ storage, and offer invaluable guidance for better carbonaceous-based anode design.

## Experimental Section

### Preparation of Graphite Oxide (GO)

Graphite oxide was prepared from graphite powder using a modified Hummers method. Briefly, 1 g of graphite powder was stirred for 1 h in 30 mL of concentrated H_2_SO_4_ in ice-bath. The required amount of KMnO_4_ (1, 3 or 5 g) was slowly added to the mixture under stirring. After vigorous stirring at 40 °C for 2 h, 60 mL of de-ionized water was added to the mixture, followed by stirring the diluted mixture at 80 °C for additional 1.5 h. The oxidation step was terminated by the addition of 80 mL of de-ionized water and 10 mL of H_2_O_2_ solution (35 wt%). With the aid of repeated centrifugation, the resulting graphite oxide suspension was washed with 5% HCl aqueous solution, and then with de-ionized water until the pH of the solution became neutral. Then the GO solution was freeze-dried to obtain dark brown GO powder (GO obtained with amount of X g KMnO_4_ is denoted as GO-X).

### Preparation of Activated Carbon

Active carbon (AC) powder (Nanjing XFNANO Materials Tech Co. C) was pre-activated by KOH. In a typical synthesis, the mixture of AC and KOH with the mass ratio of 1:5 was heated at 800 °C for 1 h in Ar atmosphere. After the reaction was completed, the obtained calcined product was washed with 1 M HCl and de-ionized water to remove excess KOH. The final AC as cathode for PIHCs was got after drying at 60 °C for 12 h.

### Material Characterizations

The morphology of graphite and as-prepared GO-X were characterized using field emission scanning electron microscopy (FESEM, Hitachi, S-4800). Fourier-transform infrared (FT-IR) spectra were collected using a Thermo Fisher Scientific, Nicolet iS50 spectrometer. The local chemical environment of the samples was characterized using an X-ray photoelectron spectrometer (XPS, Thermo Fisher-VG Scientific, ESCALAB250Xi). X-ray diffraction (XRD) patterns were recorded by Rigaku Miniflex 600 diffractometer with Cu Kα radiation (λ = 1.5406 Å). Raman spectra were performed on a confocal microscopy system using a 532 nm laser as the incident light (WITec Alpha-300R), and the specific surface area and porosity were examined by nitrogen adsorption and desorption tester collected at 77 K (JWGB, JW-BK200C).

### Electrochemical Measurements

The electrochemical properties of graphite and GO samples in PIBs were characterized by the coin-type half cells (2032) using K metal as counter electrode. The working electrode was prepared by mixing active materials, super P and polyvinylidene fluoride (PVDF) binder in a mass ratio of 7:2:1, and then dispersed in N-methyl-2-pyrrolidone (NMP) to form the homogeneous slurry. Subsequently, the uniformly mixed slurry was coated on a copper foil and dried at 80 °C in vacuum atmosphere for at least 12 h. The average areal mass loading of the active materials in each electrode was about 1 mg cm^−2^. Glass microfiber filter (Whatman GF/D) was utilized as separator. The electrolyte was 0.8 M KPF_6_ (potassium hexafluorophosphate, 99.5%) in EC (ethylene carbonate, 99%)/DEC (diethyl carbonate, ≥ 99%) (1:1, volume ratio). Cyclic voltammetry (CV) profiles were conducted on Solartron analytical system in the voltage range from 0.01 to 3.0 V. Galvanostatic charge/discharge (GCD) cycling and rate performance were performed on Land battery testing system. Electrochemical impedance spectroscopy (EIS) was obtained in the frequency range of 0.01–10^6^ Hz. PIHCs full cell was assembled using AC cathode and graphite or GO anode in 0.8 M KPF_6_ EC/DEC electrolyte with Whatman glass fiber as the separator. For fabrication of AC cathode, AC, super P, and sodium carboxymethyl cellulose (CMC) were mixed with a mass ratio of 8:1:1 in de-ionized water and coated on an aluminum foil. Before the assembly of PIHCs, the anodes were cycled at 0.1 A g^−1^ for ten cycles in half batteries. The voltage ranges of AC cathode half-cell and PIHCs full cell were 1.5 ~ 4.2 and 0.01 ~ 4.2 V, respectively. The energy density (E, Wh kg^−1^) and power density (P, W kg^−1^) of PIHCs devices were calculated based on the total active material mass of both cathode and anode (2.8 ~ 3.0 mg) as follows:

$${\text{E = }}\smallint {\text{UI dt/3}}{.6}$$

$$P = E \times 3600/\Delta t$$

where U is the working voltage (V), I is the current density (A g^−1^), and Δt is the discharging time (s).

## Results and Discussion

### Synthesis and Characterization of Graphite Oxide

**Fig. 1:**
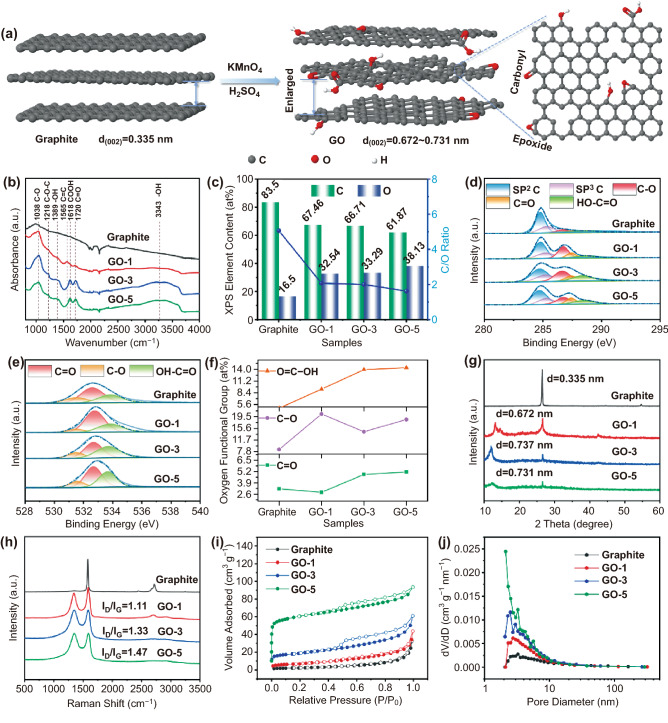
Schematic illustration of the synthesis and material characterization of graphite and GO samples. **a** Schematic illustration of the synthesis of GO. **b** FT-IR spectra. **c** XPS elements contents. **d** High-resolution C 1s spectra. **e** High-resolution O 1s spectra. **f** O element types content. **g** XRD patterns. **h** Raman spectra. **i** Nitrogen adsorption–desorption isotherms. **j** Pore size distribution

Graphite powders are functionalized with oxygen groups by the modified Hummers method to obtain GO, as-depicted in Fig. 1a. GO samples with different degrees of oxidation are obtained by adjusting the quantity (1, 3, and 5 g) of KMnO_4_ oxidizing agent. The as-obtained products are denoted as GO-1, GO-3, and GO-5, respectively. The effects of oxidation treatment on morphologies were characterized by field emission scanning electron microscope (FESEM) as shown in Figs. S1 and S2. The pristine graphite (Fig. S1) presents large flakes with a size in the range of 6 ~ 10 μm. After oxidation treatment (Fig. S2), a higher degree of exfoliation is evidenced when increasing the amount of KMnO_4_. At a low oxidant content (1 g), partial graphite begins to be exfoliated, while the high oxidant addition (5 g) breaks the few-layers graphite flakes due to excessive oxidation. With 3 g of KMnO_4_ addition, GO-3 displays regular few-layers graphite flakes structure, which is expected to facilitate the transport of K^+^.

The existence of abundant oxygen-containing groups in GO samples is confirmed by FT-IR spectroscopy, as shown in Fig. 1b. All samples exhibit overlapping bands in 1950–2200 cm^−1^ range corresponding to the diamond crystal of attenuated total reflectance (ATR) mode. Comparing to the pristine graphite, new peaks appeared in GO samples, corroborating the presence of ethers and/or epoxides (C–O–C, 1038 and 1218 cm^−1^), hydroxyls (OH, 1389 cm^−1^ and broad peak at 3343 cm^−1^), carboxyls (COOH, 1618 cm^−1^), and carbonyls (C = O, 1720 cm^−1^) [25, 26]. The peak locating at 1565 cm^−1^ is a resonance peak that can be assigned to the C = C stretching in GO. As expected, the peak intensities of the COOH and C = O bonds increase substantially from GO-1 to GO-5, implying the increase in oxidization content.

The X-ray photoelectron spectroscopy (XPS) (Figs. 1c and S3) was performed, in a next step, to corroborate the presence of the aforementioned oxygen functional groups and meanwhile provide quantitative analysis. From the survey scans shown in Fig. S3, all samples are predominantly composed of C and O elements. The surface oxygen content increases from 16.5 at% (raw graphite) to 32.5, 33.3, and 38.1 at% for GO-1, GO-3, and GO-5, respectively. And the C/O ratio decreases from 5.1 (pristine graphite) to 1.6 (GO-5). The high-resolution XPS spectrum of C 1 s in Fig. 1d exhibits five peaks at 284.7, 285.2, 286.7, 287.4, and 288.6 eV, corresponding to C–C (sp^2^), C–C (sp^3^), C–O, C = O, and COOH bonds, respectively [27]. The high-resolution spectrum of O 1s is shown in Fig. 1e, where three peaks centered at 531.5, 532.7, and 533.7 eV are corresponding to three kinds of oxygen-containing functional groups, C = O, C–O (O–C–O and C–OH), and COOH, respectively. Quantitative analysis reveals the relative ratio of C = O, C–O, and COOH varies from samples (Fig. 1f). Specifically, the total content of C = O and COOH shows the same trend as the amount of added KMnO_4_, in the order of graphite (7.9 at%), GO-1 (12.1 at%), GO-3 (18.8 at%), GO-5 (19.5 at%). Besides, among GO samples, the GO-1 delivers the highest C–O content (20.4 at%) while the lowest value is identified for GO-3 (14.5 at%). This is probably due to (1) the fact that C–O locating at the basal plane and/or the edge of GO is more preferentially to be formed with respect to C = O and COOH, especially in case of low KMnO_4_ content, and (2) the high oxidant environment drives the formation of C = O and COOH. The aforementioned results highlight that the oxidation degree and the distribution of different kinds of oxygen functional groups can be well tuned by the addition of KMnO_4_.

Evident from X-ray diffraction patterns (Fig. 1g), the (002) plane characteristic peaks of GO are clearly identified at 13.2 for GO-1, 12.0 for GO-3, and 12.1 for GO-5, respectively, in addition to the typical graphitic peak at 26.6. The corresponding interlayer spaces are determined subsequently, following an increase trend, that is, 0.335 nm (Graphite) < 0.672 nm (GO-1) < 0.737 nm (GO-3) ≈ 0.731 nm (GO-5). The enlarged interlayer space is favorable for K^+^ diffusion and maintaining electrode structural integrity.

Raman spectroscopy analysis further elucidates the structural change of graphite upon increasing KMnO_4_ oxidizing agent (Fig. 1h). D-band vibration (1345 cm^−1^) of disordered *sp*^3^-hybridized carbon atoms at defects and disordered sites in the graphitic layers, are clearly identified for GO samples, except for the G-band peak centered at 1587 cm^−1^. This demonstrates the introduction of defective surface domains including oxygen substitutional dopants, aliphatic fragments, or vacancies (i.e., pentagons or heptagons). Moreover, the I_D_/I_G_ intensity ratios increase from 1.11 for GO-1 to 1.33 for GO-3, and 1.47 for GO-5, respectively, indicating the increased disorder degree with the increase of KMnO_4_ oxidant. The specific surface areas are determined to be 8.3, 24.8, 69.3, and 226.5 m^2^ g^−1^ for graphite, GO-1, GO-3, and GO-5, respectively (Fig. 1i). The increase trend of specific surface area agrees with the increase in disorder degree as discussed above. Pore size distributions from 2 to 10 nm are also identified (Fig. 1j), corroborating the mesopore feature of GO samples. These features in terms of enlarged interlayer space, disorder structures, and abundant mesopores, are particularly beneficial for efficient K^+^ storage.

### Enhanced K^+^ Storage Performance in PIBs via Introducing Oxygen-Containing Functional Groups

The electrochemical K^+^ storage performance of the graphite and GO materials is systematically investigated and compared (Figs. S4 and 2). From cyclic voltammetry curves (Fig. S4a), a pair of well-defined redox peaks at 0.40/0.04 V is identified for graphite, characteristic of reversible K^+^ de-/intercalation process. The weak and broad peak centered at 0.67 V appears during the first cathodic scan and disappears in the subsequent cycles, which could be ascribed to the decomposition of electrolyte and the formation of SEI. However, GO samples behave differently in the aspect of CV curves (Fig. S4b–d). There are no redox peaks, and the CV curves are more like rectangular shape. These differences are further manifested by galvanostatic charge/discharge (GCD) profiles (Fig. S5) and the differential capacity (dQ/dV) profiles (Fig. S6). Upon the first discharge process, a slope region from 1.0 to 0.4 V and two plateau regions at 0.25 and 0.16 V can be observed for graphite, while the latter is absent in GO samples. Three pairs of redox peaks located at 0.50/0.36, 0.40/0.23, and 0.29/0.16 V are well identified from the dQ/dV curves of graphite, corresponding to the step transformation processes of K-graphite intercalation compound (K-GIC) [28], in good agreement with the reported graphite in PIBs [3]. For GO samples, only a broad anodic peak at about 0.64 V is detected. These differences in electrochemical behavior can be rationally attributed to the different K^+^ storage mechanisms. Generally, the energy storage mechanism of K^+^ in carbonaceous materials can be divided into two types: adsorption/desorption of K^+^ on the surface of carbon layers and de-/intercalation of K^+^ between carbon layers. The former is manifested by the sloping charge/discharge profiles and broaden redox peaks, and the latter is characteristic of well-defined plateaus in GCD curves, and oxidation/reduction peak pairs in CV curves. Thus, the above results indicate that the surface adsorption/desorption is dominant for GO samples, while the K^+^ de-/intercalation dominates in graphite sample. The introduction of abundant oxygen-containing functional groups provides more active sites for K^+^ storage. Moreover, the enlarged interlayer space induced by the introduction of these functional groups also enhances the K^+^ de-/insertion kinetics.

**Fig. 2:**
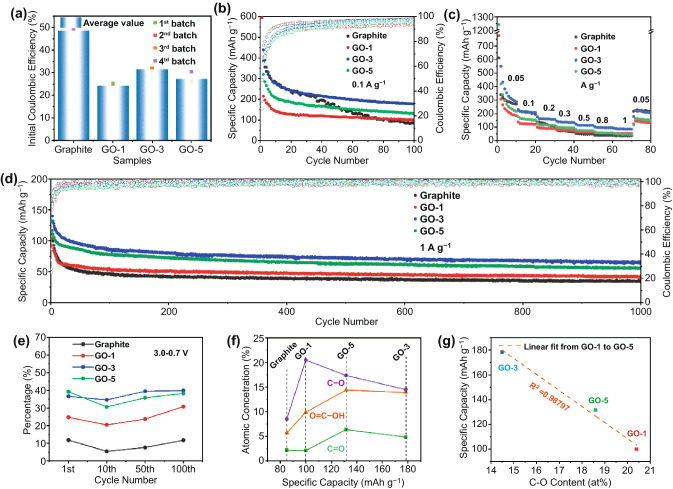
Electrochemical properties of graphite and GO samples. **a** Initial Coulombic efficiency. **b** Cycling performance at 0.1 A g^−1^. **c** Rate performance. **d** Long-term cycling performance at 1 A g^−1^. **e** Percentage of discharge capacity delivered in the voltage range of 3.0–0.7 V. **f** Relationship between atomic concentration of various oxygen functional groups obtained from O 1s XPS spectra (Fig. 2 h) and reversible specific capacity after 100 cycles at 0.1 A g^−1^. **g** Relationship between reversible specific capacity and C-O content

At a low current density of 0.1 A g^−1^, the average initial Coulombic efficiencies (ICE, Fig. 2a), determined by four batteries are found to decease in the order of graphite (48.7%) > GO-3 (31.5%) > GO-5 (27.1%) > GO-1 (24.2%), and there is no straightforward correlation between ICE and specific surface area. Interestingly, we observe a negative relationship between ICE and the C-O content. This suggests that it is the oxygen functional groups, rather than the specific surface area, that dominates the ICE. It is probably because the oxygen functional groups take part in the formation of SEI layers, and more evidences and/or discussions will be provided later. Figure 2b further compares the cycling performance and Coulombic efficiencies (CE) of these samples. High reversible capacities of 178.3 mAh g^−1^ for GO-3, 131.5 mAh g^−1^ for GO-5, and 99.9 mAh g^−1^ for GO-1, are obtained after 100 cycles, which are all above the capacity of 85.1 mAh g^−1^ for graphite. Moreover, GO-3 exhibits better rate performance within the current density range of 0.05–1 A g^−1^ (Fig. 2c). Specifically, the GO-3 electrode delivers the highest average reversible capacities of 328.6, 214.5, 161.1, 132.0, 113.5, 96.9, and 87.1 mAh g^−1^ under the current densities of 0.05, 0.1, 0.2, 0.3, 0.5, 0.8, and 1 A g^−1^, respectively. On the contrast, graphite, GO-1, and GO-5 deliver lower capacities at all rates. Although graphite shows higher capacity below 0.3 A g^−1^, the capacities of GO-1 and GO-5 exceed those of graphite at high rates (0.3–1 A g^−1^), confirming the superior kinetics thanks to the oxygen functional group-induced surface capacitance. Figure 2d shows the long-term cycling performance of the graphite and GO at 1 A g^−1^. The capacities are retained to be 63.6, 55.2, and 40.9 mAh g^−1^ after 1000 cycles for GO-3, GO-5, and GO-1, respectively, higher than that of graphite (35.3 mAh g^−1^). Detailed comparison show that the electrochemical performance is in the order of GO-3 > GO-5 > GO-1 > graphite. The ever increasing I_D_/I_G_ values and specific surface area of the GO samples, as well as the similarity of the interlayer spacing of GO-5 to that of GO-3, suggest that the differences in GO sample performance are not linearly related to graphitization structure, defects, specific surface area or interlayer spacing. Thereby, the importance of the content and type of oxygen species in determining the total reversible capacity is evidenced.

### Establishing the Relationship of Various Oxygen-Containing Functional Groups with the Performance Enhancement

Due to the superior rate capacity and excellent cycling performance of GO-3 over the others, it is necessary and meaningful to find out the relationship between oxygen functional group content/type and electrochemical properties. As shown in Fig. S7, the selected GCD profiles at 0.1 A g^−1^, i.e., 1^st^, 10^th^, 50^th^, and 100^th^ cycles, are divided into two regions: the surface (3.0–0.7 V) and diffusion-controlled (< 0.7 V) capacity contribution. This is supported by in situ Raman spectroscopy and the detailed discussion is shown in Figs. S8 and S9. It is clear that the capacitance contribution in GO is higher than that in graphite, which again confirms that the oxygen functional groups react with K^+^ (Fig. 2e). In all samples, a decreased proportion of the capacity delivered in the 3.0–0.7 V region is evidenced in the 10^th^ cycle, which is due to the reduction of irreversible side reactions between electrode and electrolyte. Upon cycling, a reversed trend is observed, indicating that the reaction of oxygen functional groups with K^+^ has higher reversibility and stability than the de-/intercalation process. It is worth noting that the amount of oxygen functional group in GO-3 is lower than that of GO-5 and close to that of GO-1. The highest reversible capacity (Fig. 2b) and the high capacity contribution in the range of 3.0–0.7 V of GO-3 (Fig. 2e) indicate that the type of oxygen functional group rather than the amount plays a critical role on the electrochemical performance. In Fig. 2f, the atomic contribution of individual oxygen functional group and reversible capacity (0.1 A g^−1^ at 100^th^ cycle) is deconvoluted. We find a strong negative correlation between the reversible capacity and C-O content with an R^2^ value of ~ 0.99 (Fig. 2g). Therefore, the above results unambiguously support the benefits of the C = O and COOH. Given that GO-5 has similar layer spacing and larger specific surface area than GO-3, but lower reversible capacity than that of GO-3, it is reasonable to conclude that the type of oxygen functional group rather than the layer spacing or specific surface area dominates the reversible capacity enhancement. This finding provides guidance for oxygen doping to improve K^+^ storage performance.

### In-depth Investigations of the Impact of Various Oxygen-containing Functional Groups on the Electrode/Electrolyte Interfacial Properties

**Fig. 3:**
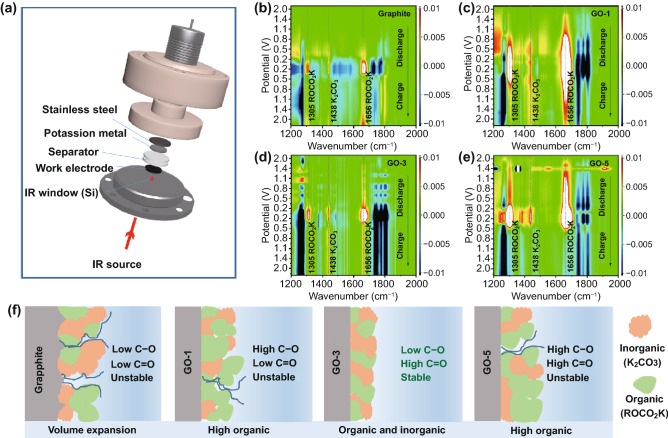
In situ FT-IR analysis. **a** Schematic representative of the in situ FT-IR spectroelectrochemical cell. FT-IR spectra of **b** Graphite, **c** GO-1, **d** GO-3, **e** GO-5. **f** Schematic diagram of the SEI components

**Fig. 4:**
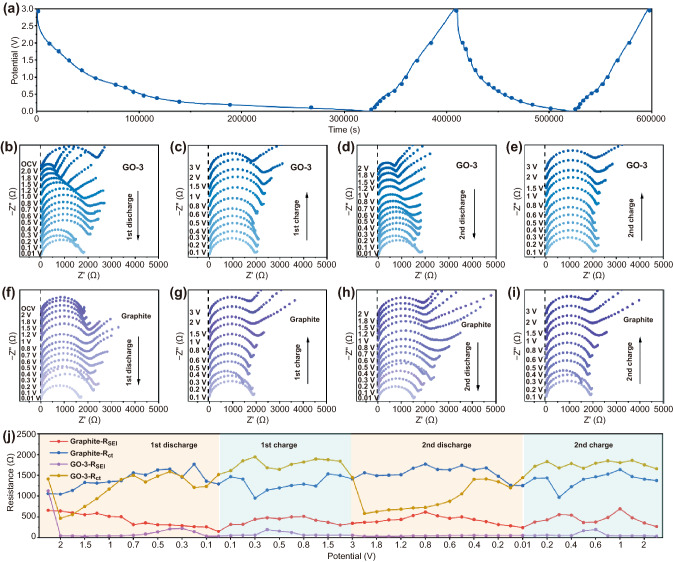
In situ kinetic diagnosis during charge and discharge of graphite and GO-3. **a** GCD profile of GO-3 during the in situ EIS measurement. **b–e** Nyquist plots of GO-3 at different potentials during GCD processes. **F–i** Nyquist plots of graphite at different potentials during GCD processes. **j** Corresponding impedances at different potentials during the GCD processes

The in situ FT-IR characterization was carried out to study the electrode/electrolyte interfacial property via tracking the SEI formation and/or evolution of GO and graphite electrodes. A schematic representative of the in situ FT-IR cell is shown in Fig. 3a. A trapezoidal Si crystal with a measurable frequency range of more than 1100 cm^−1^ is used as an IR window with a mercury cadmium telluride (MCT) detector. The working electrode coated on a porous carbon paper current collector is directly contacted with the Si crystal. Two sheets of glass filter are used as a separator, and a K metal sheet supported by a stainless-steel disk is used as the counter electrode, respectively. Based on this in situ cell device, the SEI formation and solvent change process of the electrode/electrolyte interface during the charge and discharge process can be visualized by in situ FT-IR. Figures 3b–e and S10 show the potential dependence of in situ FT-IR spectra of GO and graphite electrodes, during the initial discharge/charge cycle. The corresponding GCD curve is shown in Fig. S11. FT-IR spectra of the neat electrolyte and solvent were also collected as reference. More details about peak assignment are provided in Supporting Information (Fig. S12 and Table S1). All in situ FT-IR spectra were subtracted from the FT-IR profile at OCP to clarify the changes in the reflection spectra during charging and discharging. In the subtractive spectra, positive and negative peaks correspond to the vibration of the newly formed and decreased species. Three new peaks locating at 1305, 1438, and 1661 cm^−1^ appear in the spectra of GO electrodes at ~ 2.0 V (Figs. 3c–e and S10b-c), accompanying with the decomposition of EC (downward peaks at 1772 and 1799 cm^−1^) and DEC (downwards peak at 1275 and 1741 cm^−1^). These new peaks are assigned to potassium alkyl carbonate (ROCO_2_K) (1305 and 1661 cm^−1^) and K_2_CO_3_ (1438 cm^−1^), respectively. The potential of ~ 2 V is just the electrolyte reduction potential as evident from in situ EIS results (Fig. 4), and marks the start of SEI formation on GO electrodes. New features are also observed in the spectra of graphite electrode (Figs. 3b and S10a), but in a much lower potential range (~ 0.8 V) with respect to GO electrodes. The difference in electrolyte reduction potential corroborates that the oxygen functional groups, act as catalysts that promoting the solvent’s decomposition and the formation of the intact SEI at a much higher potential [29]. Note that the positive peaks intensity of the spectra in GO samples are much stronger than that of graphite, demonstrating that the SEI layer formed on GO electrodes is much thicker than the graphite one [30]. The thickness of SEI is estimated to decrease in the order of GO-1, GO-5, GO-3, and graphite, reflecting the increasing trend of ICE, which is consistent with the experimental result (Fig. 2a). The thin SEI cannot form a good protection for the large volume expansion of the graphite electrode, proved from the cross-sectional FESEM images (Fig. S13). The volume expansion (at the 10th fully charged state) are calculated to be 12.0% for GO-1, 5.5% for GO-3 and 6.7% for GO-5, which are much smaller than that of graphite electrode (30.8%). Detailed analysis reveals that the reduction selectivity is also strongly dependent on electrode types. Specifically, more ROCO_2_K are identified for GO-1 with a much wider potential distribution, followed by GO-5, GO-3 and graphite. This trend is in line with the decrease order of C-O content, indicating that C-O group favors the formation of ROCO_2_K. On the contrast, C = O and COOH facilitate the formation of K_2_CO_3_, evident from Figs. 3b–e and S10. More K_2_CO_3_ is produced in the GO-3 derived SEI, followed with GO-5, GO-1 and graphite. K_2_CO_3_ is superior over ROCO_2_K in the formation of intact, stable and highly ionic conductive SEI, benefiting from less gaseous products involved [31]. This well explain the better rate capability and cycling stability of GO-3. In addition, the weaker peak intensity of both K_2_CO_3_ and ROCO_2_K in GO-3 also suggest that the GO-3 derived SEI is much thinner compared to GO-1 and GO-5. The schematic diagram of the initial SEI formation (shown in Fig. 3f) is provided to explain the selectivity surface dependence features. In summary, C-O significantly promotes the production of ROCO_2_K, and C = O (C = O and COOH) facilitates the generation of K_2_CO_3_. GO-3 with the lowest C-O content and high C = O (C = O and COOH) content exhibits intact SEI layer with a proper ratio of organic and inorganic components that is electrochemical stable, intact and highly ionic conductive. High conductivity SEI of GO-3 will be confirmed by the EIS test discussed below. The related peak intensities of ROCO_2_K and K_2_CO_3_ gradually weakened upon charging without complete disappear. This corroborates the dynamic behavior of SEI and it remains stable for protecting the electrode integrity in the following cycles.

It has been reported that although graphene oxide with oxygen functional groups raises the electrode impedance, it still exhibits higher specific capacitance than graphene, indicating that the lower electrode conductivity does not impede its application in energy storage [20]. Similarly, in this work, GO samples with oxygen functional groups exhibit better rate capability and cycling stability than graphite. Therefore, the charge transfer kinetics and SEI evolution upon charging and discharging process of graphite and GO-3 electrode system are further investigated using in situ EIS. Figures 4a and S14a exhibit the GCD profiles of initial two cycles acquired from in situ EIS measurement at 20 mA g^−1^. To ensure the quasi-equillibrium state, the in situ EIS was recorded when the battery was held at a given potential for 30 min. All Nyquist plots (Fig. 4b–i) are comprised of a depressed semicircle in the high/medium frequency region corresponding to SEI resistance (R_SEI_) and charge transfer resistance (R_ct_), and a sloping line in the low frequency range assigning to Warburg impedance of ion diffusion. The intersection of the EIS curve with the real axis is attributed to ohm resistance (R_Ω_), which mainly reflects the resistance of electrode, electrolyte and separator. The Nyquist plots are fitted using an equivalent circuit model (Fig. S15). Figures 4j and S14b quantified and summarized the impedances in different states. As depicted in Fig. S14b, the R_Ω_ is nearly independent of the state of charge (SOC) and depth of discharge (DOD). Overall, the average R_Ω_ of graphite and GO-3 are calculated to be 6.7 and 8.8 Ω, respectively. The higher R_Ω_ indicates that the introduction of oxygen functional groups increases the internal resistance of the electrode. By tracking the R_SEI_ at various SOC, the evolution of the SEI is observed. In the graphite system, the R_SEI_ decreases upon K^+^ intercalation and then increases upon K^+^ de-intercalation. Specifically, in the high potential range above 1.2 V, R_SEI_ stays at a high level of 650 ~ 550 Ω and slowly decreases as the voltage drops. R_SEI_ value sharply decreases from 586.3 to 315 Ω in a narrow voltage range of 1.2–0.7 V, corresponding to the formation of SEI. Below 0.7 V, the R_SEI_ is maintained at a low value (149.7 Ω at 0.01 V) indicating the formation of conductive SEI. However, when the GO-3 is employed as anode, the R_SEI_ decreases dramatically from 1127 Ω at OCP to 48.8 Ω at 2 V, indicating that the oxygen functional group induces the generation of highly conductive SEI at high potentials. The R_SEI_ remains at a low impedance value of 33 ~ 48 Ω during the subsequent discharge process. It is worth noting that there is only a slight increase of R_SEI_ observed upon charge process, which is 58.3 Ω at full charge to 3 V. In a sharp contrary, the R_SEI_ of graphite reversibly increases to 345 Ω in the fully charged state, suggesting that the SEI on graphite is disrupted by large volume changes due to the removal of K^+^, which has been confirmed in FESEM (Fig. S13). The low R_SEI_ value in the second cycle further confirms that the GO-3 sample develops stable and highly ionic conductive SEI layer. On the other hand, the R_ct_ of GO-3 also drops sharply from 1413 (OCP) to 466.9 Ω (2 V), which can be attributed to the activation of the electrode material. During the discharge process, GO-3 has a lower R_ct_ than graphite at high potentials above 0.1 V, and higher R_ct_ than graphite below 0.1 V. This is because the diffusion-controlled charge transfer process is limited by the higher R_Ω_ of the GO-3 electrode, and the higher R_Ω_ further leads to the higher R_ct_ of GO-3 than that of graphite during charging. Further recording of EIS during the second cycle reveals that R_ct_ evolution is a reversible process. The lower and more stable R_SEI_ of GO-3 is attributed to the regulation of SEI components and good structural stability by optimzing oxygen functional groups, as confirmed by in situ FT-IR (Fig. 3).

To examine the SEI and charge transfer evolution upon long-term cycling, EIS spectra of the graphite, GO-1, GO-3, and GO-5 electrodes at fresh and fully charged state over 100 cycles were recorded. Selected Nyquist plots are shown in Fig. S16a–d. According to the fitting result (Table S2), graphite cell exhibits the lowest overall impedance among the four samples in the fresh state, indicating that the oxygen-containing functional groups raise the impedance of the GO electrodes, consistent with the previously reported carbons [32]. The K^+^ diffusion coefficients (D_K_, cm^2^ s^−1^) of fresh graphite is 1.8 × 10^–13^ cm^2^ s^−1^, which is also higher than those of fresh GO samples (8.3 × 10^–13^ cm^2^ s^−1^ for GO-1, 2.2 × 10^–13^ cm^2^ s^−1^ for GO-3 and 2.4 × 10^–14^ cm^2^ s^−1^ for GO-5), indicating that the inactivated GO electrode has rather sluggish ion diffusion ability (Fig. S16e). However, R_SEI_ and R_ct_ of GO-3 cell dramatically decrease from 1055 and 1377 Ω (fresh) to 36 and 685.8 Ω (after 1^st^ cycle), respectively. The GO-3 cell exhibits much lower R_SEI_ (52.9 Ω) and R_ct_ (1142 Ω) in the 100^th^ cycle compared to those of the graphite cell (483.9 Ω (R_SEI_) and 2380 Ω (R_ct_)). Similar trend can be observed from GO-5 and GO-1. In addition, after cyclic activation, despite of value fluctuations, the D_K_ decreases in the order of GO-3 (6.3 × 10^–13^ cm^2^ s^−1^) > GO-5 (3.8 × 10^–13^ cm^2^ s^−1^) > GO-1 (2.0 × 10^–13^ cm^2^ s^−1^) > graphite (7.3 × 10^–14^ cm^2^ s^−1^) at 100^th^ cycle. The apparently smaller R_SEI_ and R_ct_, as well as higher ion diffusion coefficient of GO-3 explain the superior rate and cycling performance over the other three samples. The impedance is generally dependent on two opposite effects: (i) the formation of highly ionic conductive SEI that decrease the R_SEI_ and R_ct_ value, and (ii) the expansion of carbonaceous electrode, resulting in an increase in impedance. For GO samples, the significant decrease in R_SEI_ and R_ct_ value upon cycling due to the former factor, which has been confirmed by in situ EIS. The latter is verified by suppressed volume expansion evidenced from the cross-sectional FESEM images (Fig. S13). These results corroborate that (i) oxygen functional groups promote the formation of intact SEI layer with highly ionic conductivity and robustness, and (ii) the enlarged interlayer spacing induced by functionalized oxygen-containing groups is efficient in buffering volume expansion (Fig. S17). Specifically, R_SEI_ value increases in the order of GO-3, GO-5, GO-1, which is in line with the evolution trend of C-O content. This implies that (i) more C–O group leads to larger R_SEI_, and (ii) C = O and COOH might be more favorable for the formation of highly ionic conductive SEI. These results also highlight the critically important role of introducing suitable oxygen functional groups in boosting K^+^ storage performance.

According to the systematic study of in situ FT-IR, combined with in situ EIS analysis, it can be seen that through optimizing the oxygen-containing groups of GO, the regulated SEI components and the suppressed volume expansion of electrodes are contributed to low R_SEI_, stable impedance changes and high K^+^ diffusion coefficient for GO-3 electrodes, which accounts for the enhanced K^+^ storage performance in GO-3. In short, the reasons for such an enhancement are described as follows: (1) the oxygen functional groups (especially C = O/COOH) expand the graphite interlayer spacing leading to faster K^+^ diffusion kinetics and suppressed volume expansion upon cycling; (2) the oxygen functional groups provide more K^+^ active sides yielding higher deliverable capacities; (3) C = O/COOH regulate the electrode/electrolyte interfacial properties achieving the formation of highly conductive, intact and robust SEI.

### Extended Application in Potassium Ion Hybrid Capacitors (PIHCs)

**Fig. 5:**
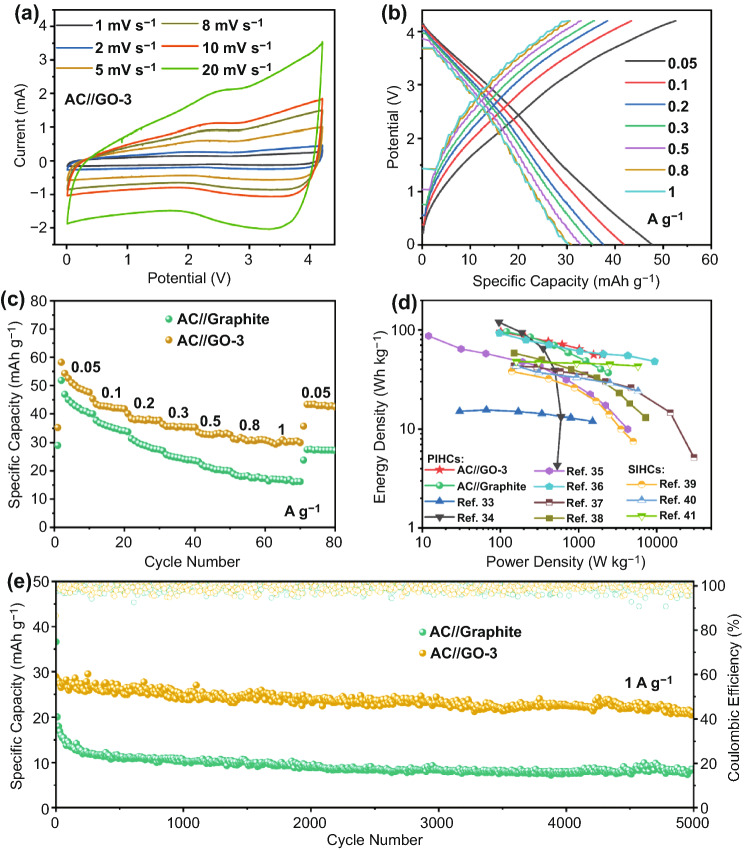
Electrochemical performance of PIHCs using graphite or GO anode and AC cathode. **a** CV curves of AC//GO-3. **b** GCD profiles of AC//GO-3. **c** Rate capability of the PIHCs. **d** Ragone plots of the PIHCs. **e** Long-term cycle performance of the PIHCs

Lastly, we further explore the application in potassium ion hybrid capacitors (PIHCs) using the GO-3 or graphite as the anode and commercial active carbon (AC) as the cathode. Before constructing the PIHCs, the AC as the cathode in half-cell was tested with metallic potassium as the counter electrode. The rate performance and charge/discharge profiles are displayed in Fig. S18. The linear charge/discharge curves of AC cathode demonstrate an adsorption/desorption mechanism for K-ion storage. The optimal mass ratio of anode to cathode is 1: 2 in the PIHCs on a basis of the charge balance theory (Q_+_  = Q_-_). The CV curves of the PIHCs are shown in Figs. 5a and S19a, with the voltage ranging from 0.01 to 4.2 V. Notably, the PIHCs do not show noticeable voltage polarization even at a high scan rate of 20 mV s^−1^, indicating excellent electrochemical stability in such a wide voltage window. In addition, the similar shapes of GCD curves (Figs. 5b and S19b) at different current densities demonstrate the excellent rate performance of PIHCs. The AC//GO-3 PIHC delivers a reversible capacity of 47.6 mAh g^−1^ at 0.05 A g^−1^ and retains 30.3 mAh g^−1^ at 1 A g^−1^ (Fig. 5c). The specific capacity of AC//GO-3 PIHCs is higher than that of AC//Graphite at different current densities. The enhanced electrochemical kinetics of GO-3 anode ensures the superior rate performance of PIHCs. The energy/power densities of the PIHCs are displayed in the Ragone plot shown in Fig. 5d. A high energy density of 96.4 Wh kg^−1^ can be achieved at 101.2 W kg^−1^. Still, an energy density of 56.1 Wh kg^−1^ is maintained even at a high power density of 1975.9 W kg^−1^, which is comparable to or even exceeds some representative PIHCs and sodium-ion hybrid capacitors [33–41]. Moreover, the AC//GO-3 PIHC exhibits a promising cycling stability with a capacity retention of 76.8% after 5000 cycles at a current density of 1 A g^−1^ (Fig. 5e), which is substantially higher than that of AC//Graphite PIHC (21.6%).

## Conclusion

In summary, GO with controlled oxygen functional groups have been successfully prepared through the modified Hummers method. It has been shown that the introduction of highly active oxygen functional groups into graphite results in a substantial increase in rate and cycling performance for PIBs, meanwhile, maintaining good structural integrity. The integration of adsorption-intercalation hybrid charge storage mechanisms of GO electrode is also demonstrated by in situ Raman. In situ EIS and FT-IR suggest that the SEI components are predominantly manipulated by the types of oxygen functional groups. It is revealed that the C-O content promotes the formation of organic component (ROCO_2_K), while the C = O and COOH enriches the formation of inorganic component (K_2_CO_3_). Systematic investigations reveal that C = O and COOH are more favorable for K^+^ storage compared to C–O and establish a clear relationship between the types/contents of oxygen functional groups and the regulated composition of SEI. The findings in this study highlight the importance of optimizing suitable types of oxygen-containing functional groups, providing guidance for the design of oxygen-doped electrode materials.

## Supplementary Information

Below is the link to the electronic supplementary material.

Supplementary file1 (PDF 2870 kb)
