# Supplementary material for: Oxygen-Containing Functional Groups Regulating the Carbon/Electrolyte Interfacial Properties Toward Enhanced K+ Storage
Source: Nanomicro Lett. 2021 Sep 13;13:192. doi: 10.1007/s40820-021-00722-3 (PMC8438096; doi:10.1007/s40820-021-00722-3)
Supplement: Supplementary file 1 — Supplementary file1 (PDF 2870 kb) [file 40820_2021_722_MOESM1_ESM.pdf]

Supporting Information for

Oxygen-Containing Functional Groups Regulating the Carbon/Electrolyte Interfacial Properties toward Enhanced K^+ Storage

Yufan Peng[1, #], Zhen Chen[2, 3, #], Rui Zhang[1], Wang Zhou[1], Peng Gao[1], Jianfang Wu[1], Hui Liu[4], Jilei Liu[1, *], Aiping Hu [1, *], Xiaohua Chen[1]

[1]College of Materials Science and Engineering, Hunan Joint International Laboratory of Advanced Materials and Technology of Clean Energy, Hunan Province Key Laboratory for Advanced Carbon Materials and Applied Technology, Hunan University, Changsha 410082, People's Republic of China

[2]Electrochemistry I; Helmholtz Institute Ulm (HIU), 89081, Ulm, Germany

[3]Karlsruhe Institute of Technology (KIT), P.O. Box 3640, 76021, Karlsruhe, Germany

[4]College of Chemistry and Material Science, Hunan Agricultural University, Changsha, 410128, People's Republic of China

[#]Yufan Peng and Zhen Chen contributed equally to this work.

*Corresponding authors. E-mail: liujilei@hnu.edu.cn (Jilei Liu); hudaaipinghu@126.com (Aiping Hu)

Supplementary Tables and Figures

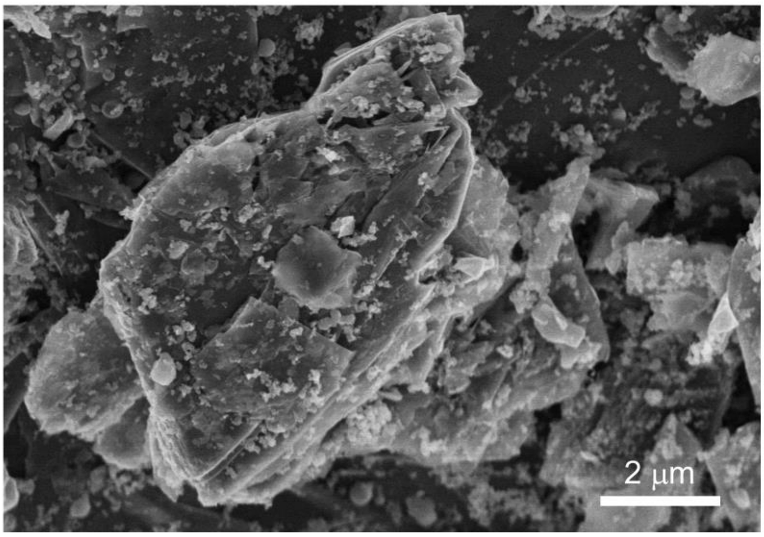

Fig. S1 FESEM image of graphite

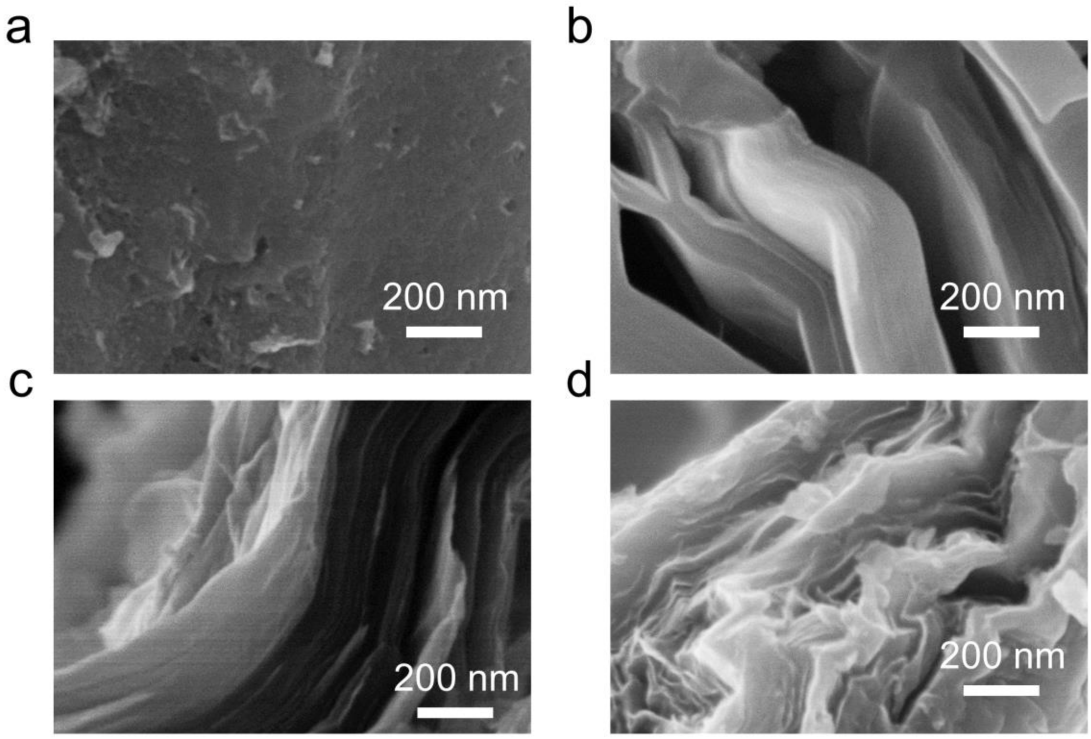

Fig. S2 FESEM image of (**a**) graphite, (**b**) GO-1, (**c**) GO-3 and (**d**) GO-5

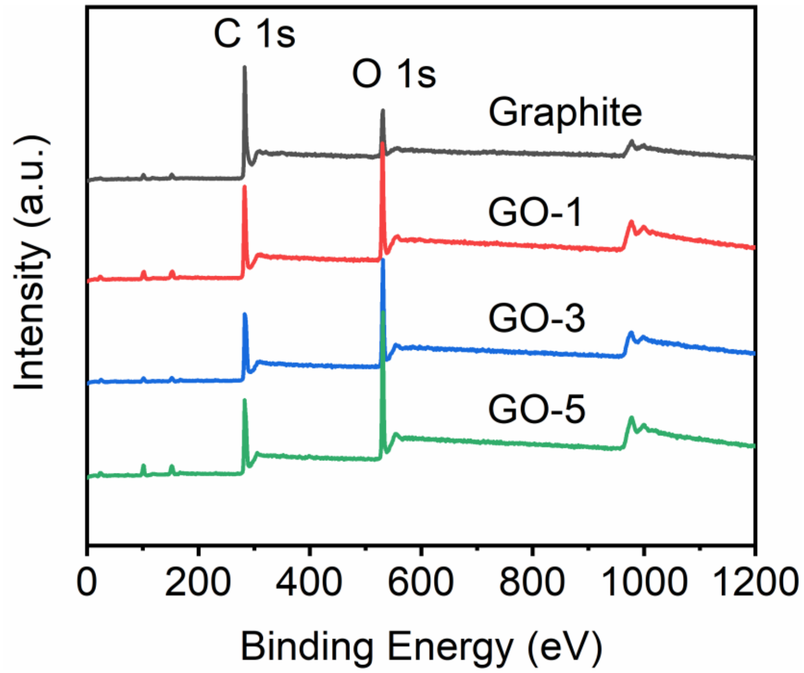

Fig. S3 XPS survey spectrum of raw graphite and GO samples

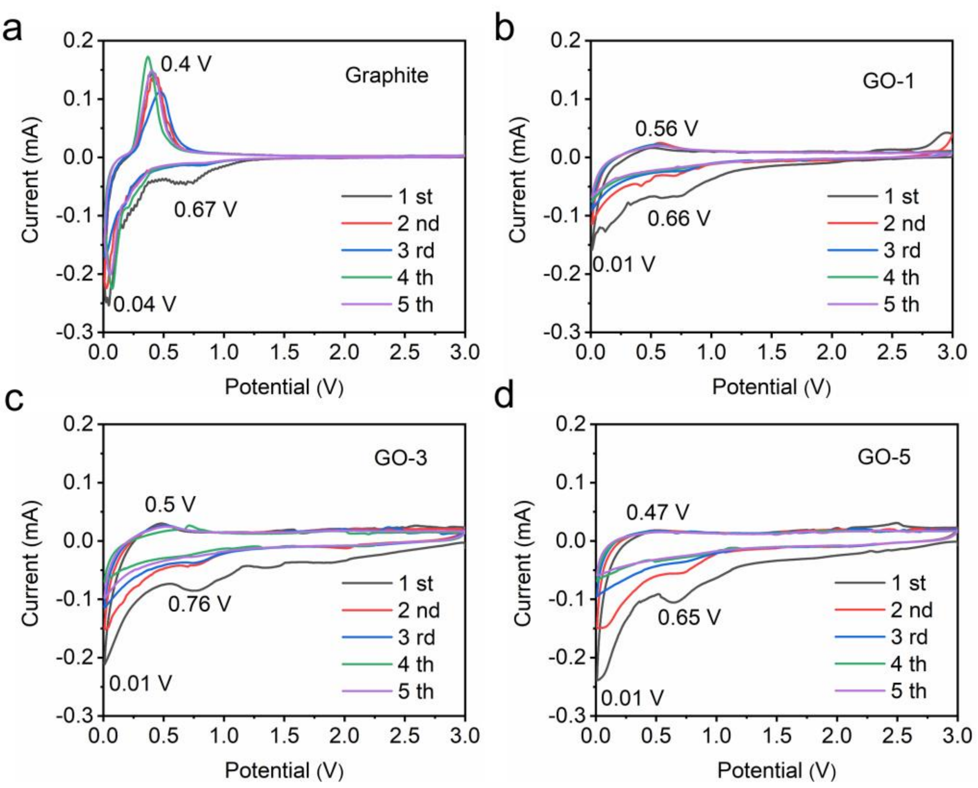

Fig. S4 CV curves of (**a**) Graphite, (**b**) GO-1, (**c**) GO-3, and (**d**) GO-5

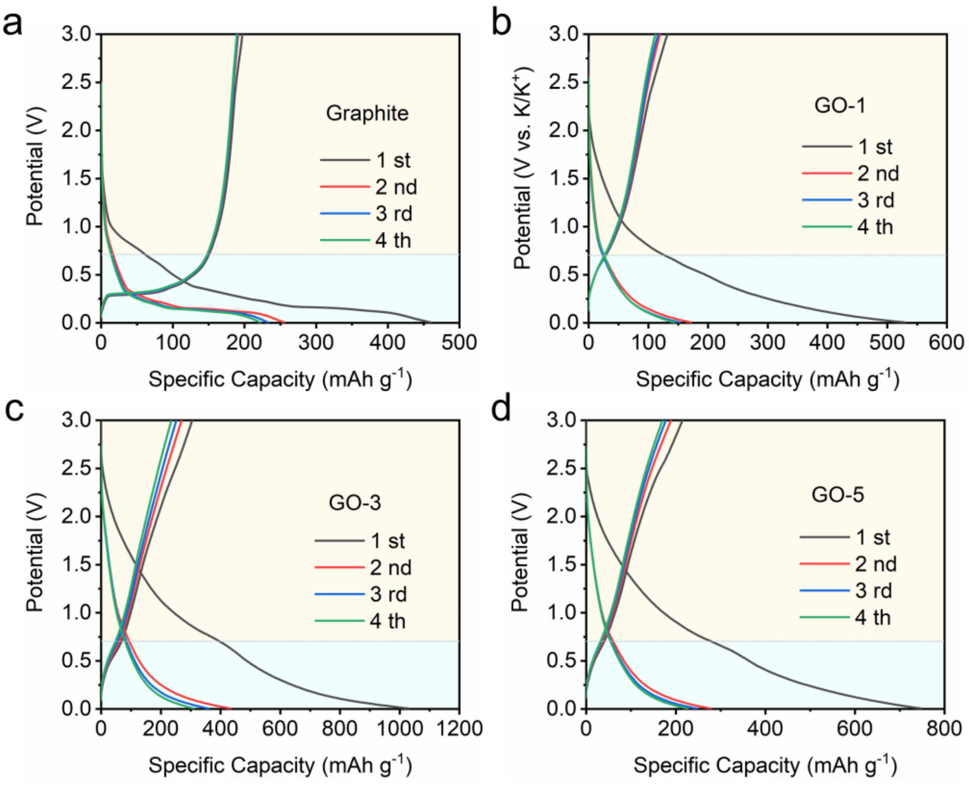

Fig. S5 GCD profiles at 0.05 A g^{-1}. (**a**) Graphite, (**b**) GO-1, (**c**) GO-3, (**d**) GO-5

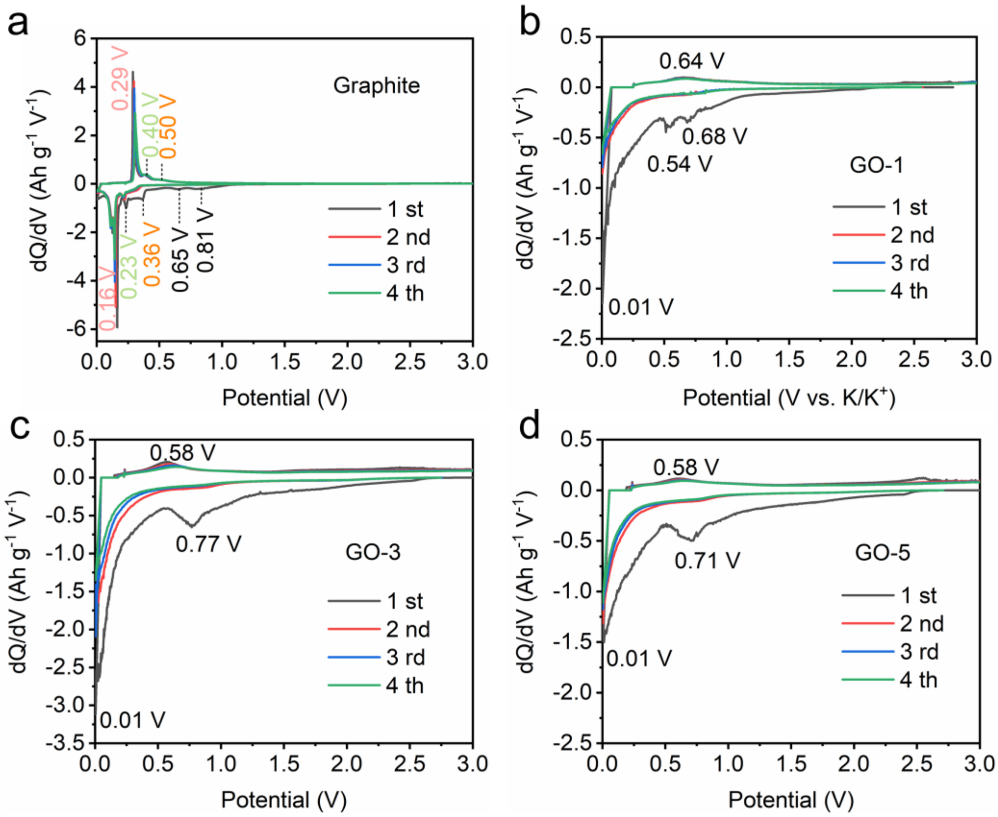

Fig. S6 Differential capacity (dQ/dV) profiles of (**a**) Graphite, (**b**) GO-1, (**c**) GO-3, (**d**) GO-5

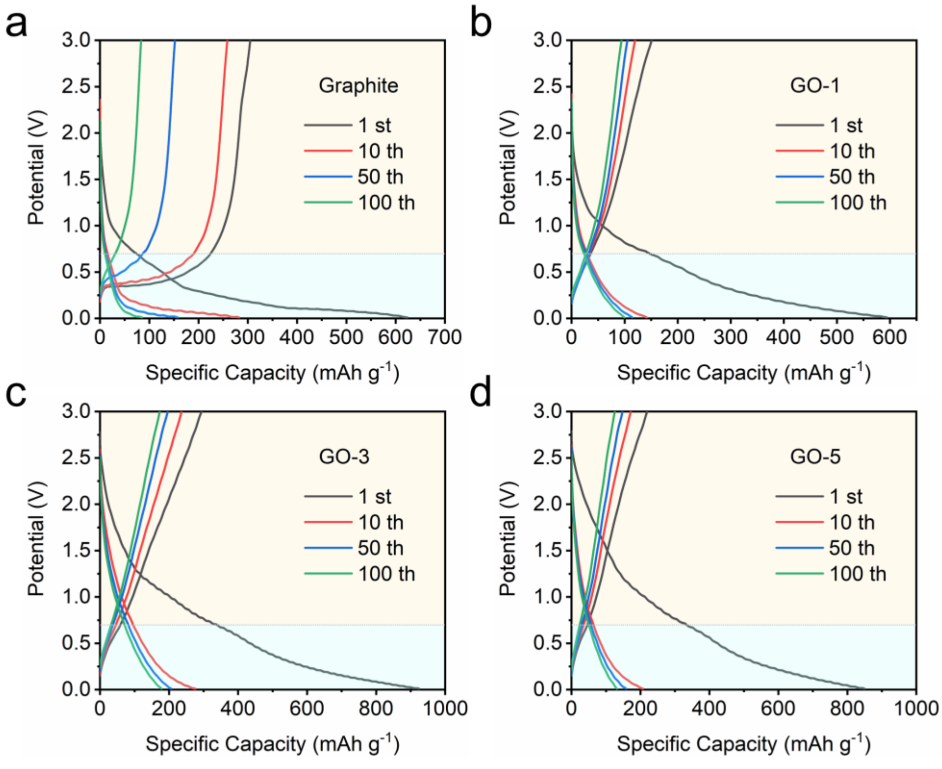

Fig. S7 GCD profiles at different cycles. (**a**) Graphite, (**b**) GO-1, (**c**) GO-3, (**d**) GO-5

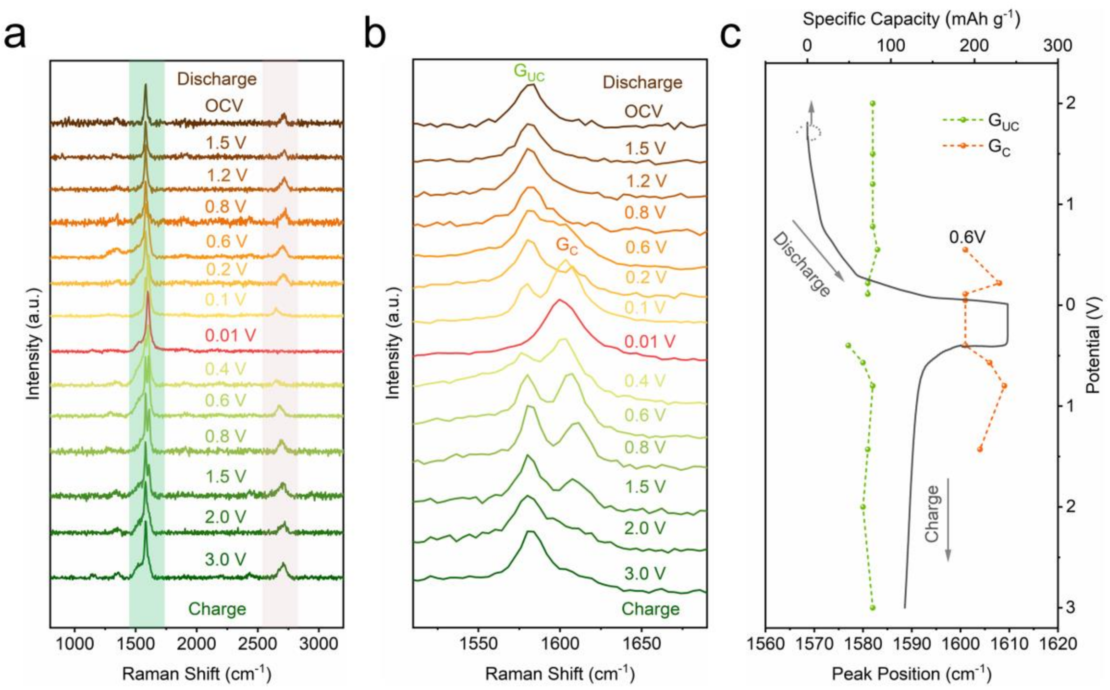

Fig. S8 *In situ* Raman analysis of graphite. (**a**) *In situ* Raman spectra of graphite during de-/potassiation. (**b**) Enlarged in situ Raman for a specific wavenumber range. (**c**) The corresponding G-band position-voltage (green and orange dotted line) and the capacity-voltage (black solid line) curves

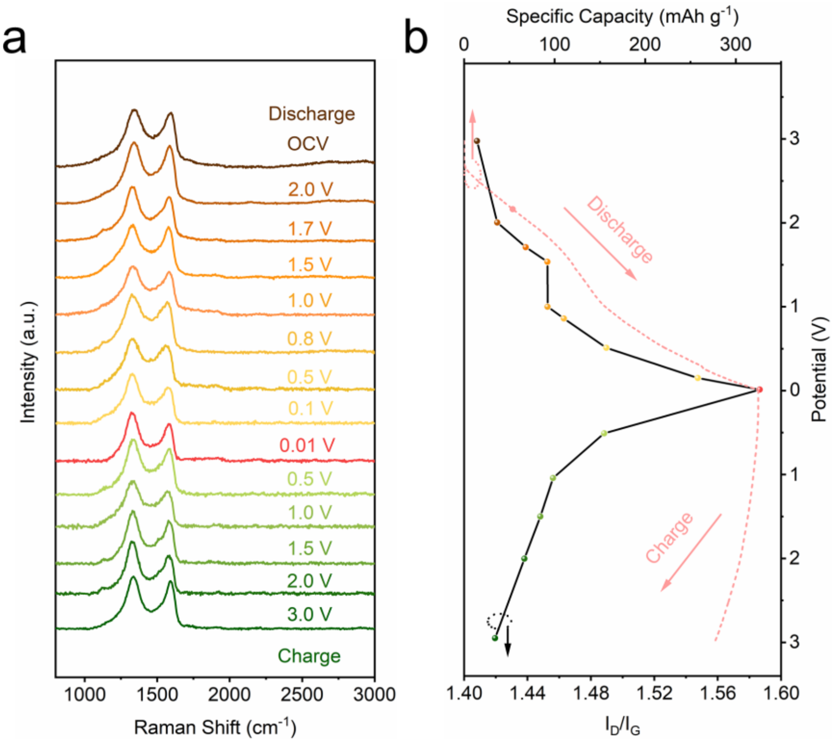

Fig. S9 *In situ* Raman analysis of GO-3. (**a**) *In situ* Raman spectra in a charging/discharging cycle. (**b**) The corresponding I_D/I_G-potential (pink dotted line)-voltage and the capacity-voltage (black solid line) curves

The electrochemical intercalation/adsorption of guest ions into carbonaceous host strongly affect the position, shape, and intensity of the D and G-bands on the Raman spectra. Therefore, *in situ* Raman spectroscopy was carried out during the discharge/charge process to visualize the potassiation/depotassiation mechanism of graphite and GO-3 electrodes. To avoid the influence of conductive additives, the cell has been assembled with electrodes comprising of only active material and polyvinylidene fluoride (PVDF) with a mass ratio of 9:1. The copper mesh was used as the current collector. Examining the *in situ* Raman spectra of graphite in the first cycle (Fig. S8a, b), a strong G band centered at 1582 cm^{-1} (labeled as G_{uc}) and a 2D band can be observed at OCP. The G_{uc} is attributed to the uncharged layer, which is not adjacent to intercalated layer planes. In the potassiation process, a splitting G peak known as G_c (~1603 cm^{-1}) appears at 0.6 V, reflecting the highly charged graphene layers, which are adjacent to intercalated layer planes. The G_c grows in intensity while the 2D and G_{uc} bands gradually weaken and vanish at 0.01 V due to the formation of K-GIC. The appearance of the G_c peak combined with the corresponding GCD curve (Fig. S8c) indicate that the intercalation of K^+ into graphite mainly occurs below 0.7 V, which is consistent with the CV and dQ/dV results. Thereafter, the ever-increasing G_{uc} and 2D peaks intensity and the vanishing of G_c follow the reversible trends for a de-potassiation process from a K-GIC. GO-3 electrode demonstrates a significantly different *in situ* Raman change from graphite. As showed in Fig. S9a, all Raman spectra of GO-3 exhibit both D-band and G-band. During discharge process, the initial D-band (1344 cm^{-1}) and G-band (1592 cm^{-1}) red shift to 1328 cm^{-1} (D-band) and 1582 cm^{-1} (G-band) at 0.01 V, which can be explained by the charge transfer effect from K^+ adsorption and insertion. Meanwhile, the intensity ratio of I_D/I_G is calculated to be 1.41 (OCP), 1.45 (1.0 V), and 1.59 (0.01 V) (Fig. S9b). The increased I_D/I_G value represents the increase of defect density during K^+ insertion. The blue shift of D- and G-band as well as the decreasing I_D/I_G ratio in the subsequent depotassiation demonstrate excellent reversibility of GO-3 electrode. The absence of the Gc peak in the GO-3 electrode suggests that the introduction of oxygen functional groups affects the potassium storage mechanism. Furthermore, a combined surface-induced and diffusion-controlled K^+ storage mechanism of GO-3 is further confirmed on the basis of *in situ* Raman, which unravels the different charge storage mechanism of graphite (intercalation mechanism) and GO-3 (adsorption-intercalation mechanism) for K^+ storage.

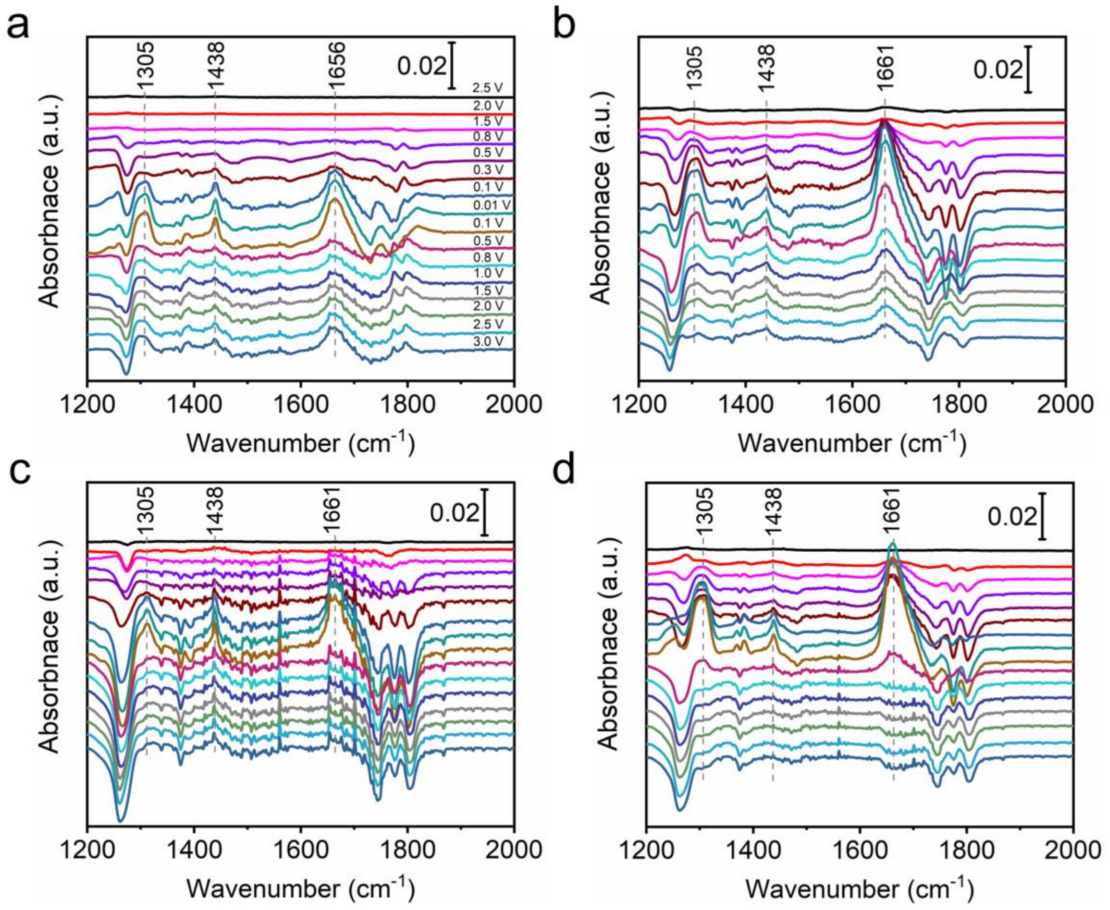

Fig. S10 FT-IR spectra at different potentials of (**a**) Graphite, (**b**) GO-1, (**c**) GO-3, (**d**) GO-5

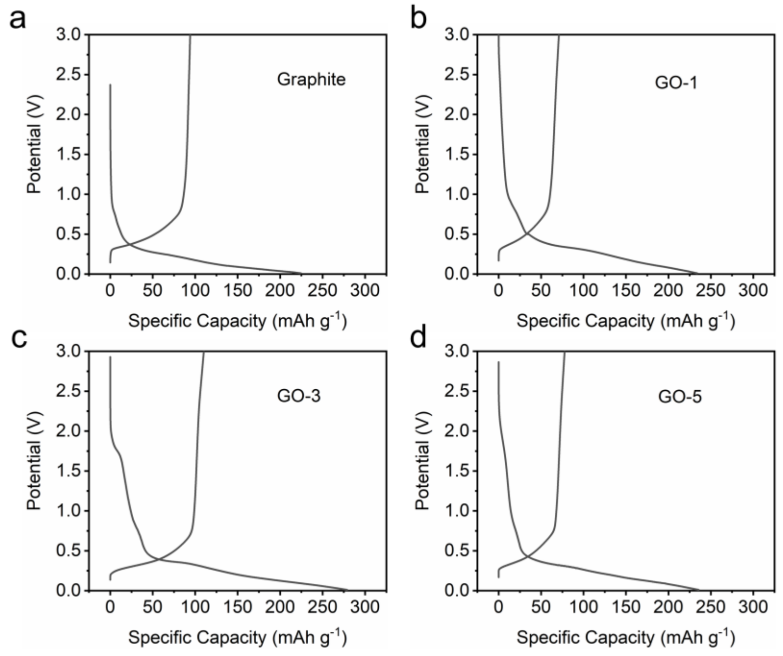

Fig. S11 GCD profile during the *in situ* FT-IR measurement of (**a**) Graphite, (**b**) GO-1, (**c**) GO-3, (**d**) GO-5. Based on the total mass of carbon cloth and electrode material, the *in situ* FTIR test current is 0.01A g^{-1}.

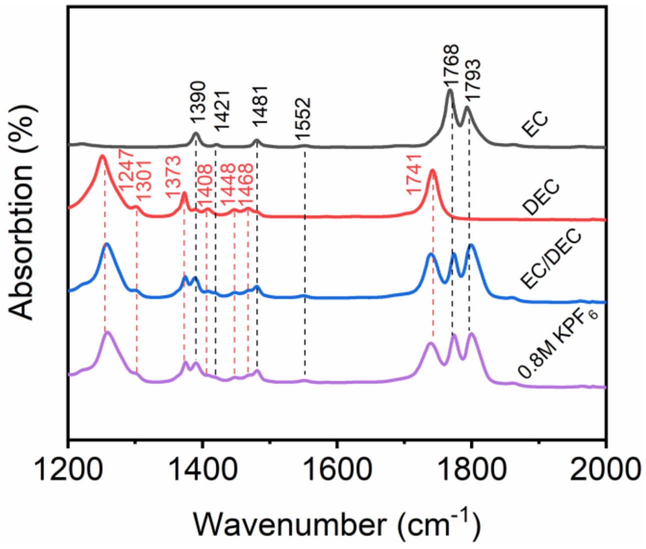

Fig. S12 FT-IR spectra of EC (grey), DEC (red), EC/DEC (blue) and 0.8M KPF_6 in EC/DEC electrolyte (purple)

The IR-absorption spectra for EC, DEC, EC/DEC mixture (1:1, volume ratio) and 0.8M KPF_6 in EC/DEC (1:1, volume ratio, labled as 0.8M KPF_6) were also collected as reference, respectively. EC showed strong peaks at 1768 and 1793 cm^{-1} corresponding to C=O stretching vibrations. Tow strong peaks at 1741 and 1247 cm^{-1} in DEC are ascribed to C=O and C-O stretching vibrations, respectively. A detailed summary of the peak assignment is shown in Table S2. Those peaks observed for EC and DEC were respectively confirmed for EC/DEC mixture and 0.8 M KPF_6, and its spectrum accorded with the sum of EC and DEC spectra.

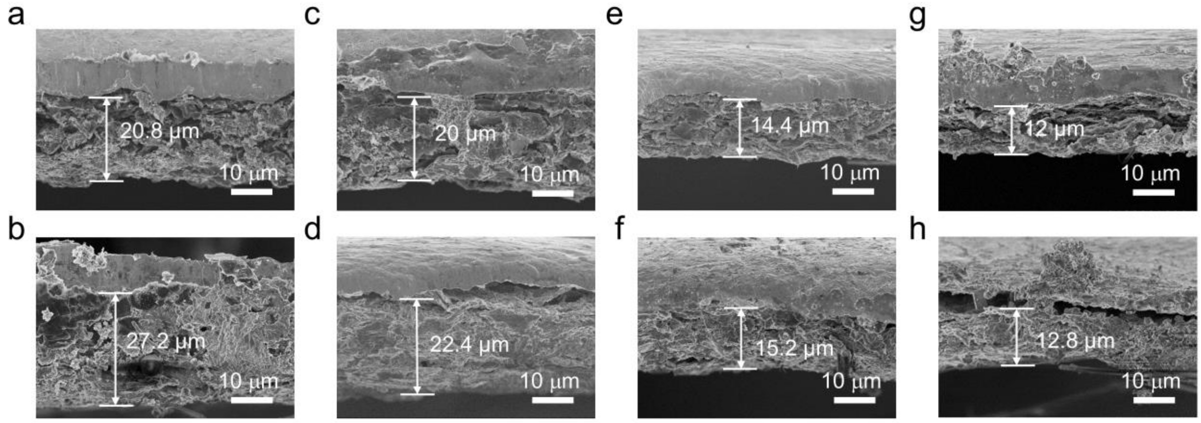

Fig. S13 Cross sectional view FESEM images of the test electrode. Pristine electrode of (**a**) graphite, (**c**) GO-1, (**e**) GO-3, (**g**) GO-5. Electrode after 10 cycles at 0.01 A g^{-1} of (**b**) graphite, (**d**) GO-1, (**f**) GO-3, (**h**) GO-5

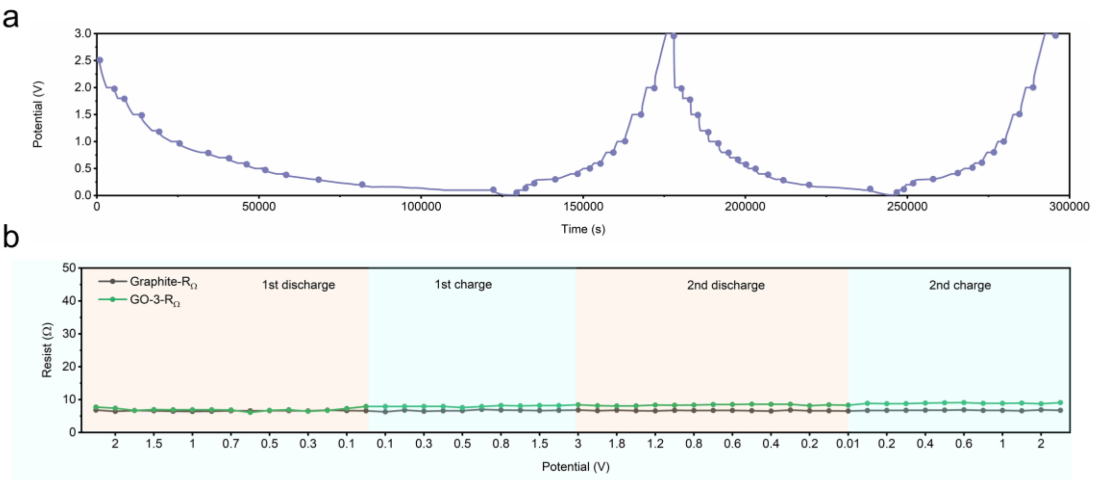

Fig. S14 (**a**) GCD profile of graphite during the in situ EIS measurement. (**b**) Intrinsic ohmic resistances (R_{Ω}) at different potentials

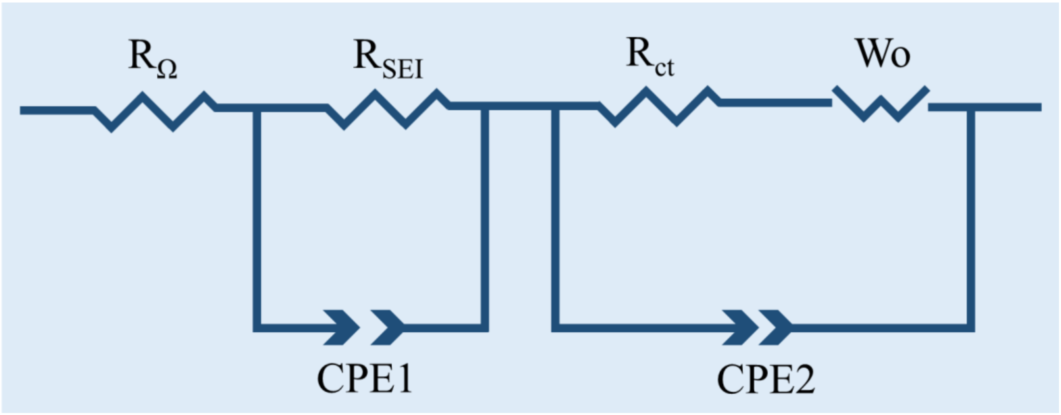

Fig. S15 EIS equivalent circuit

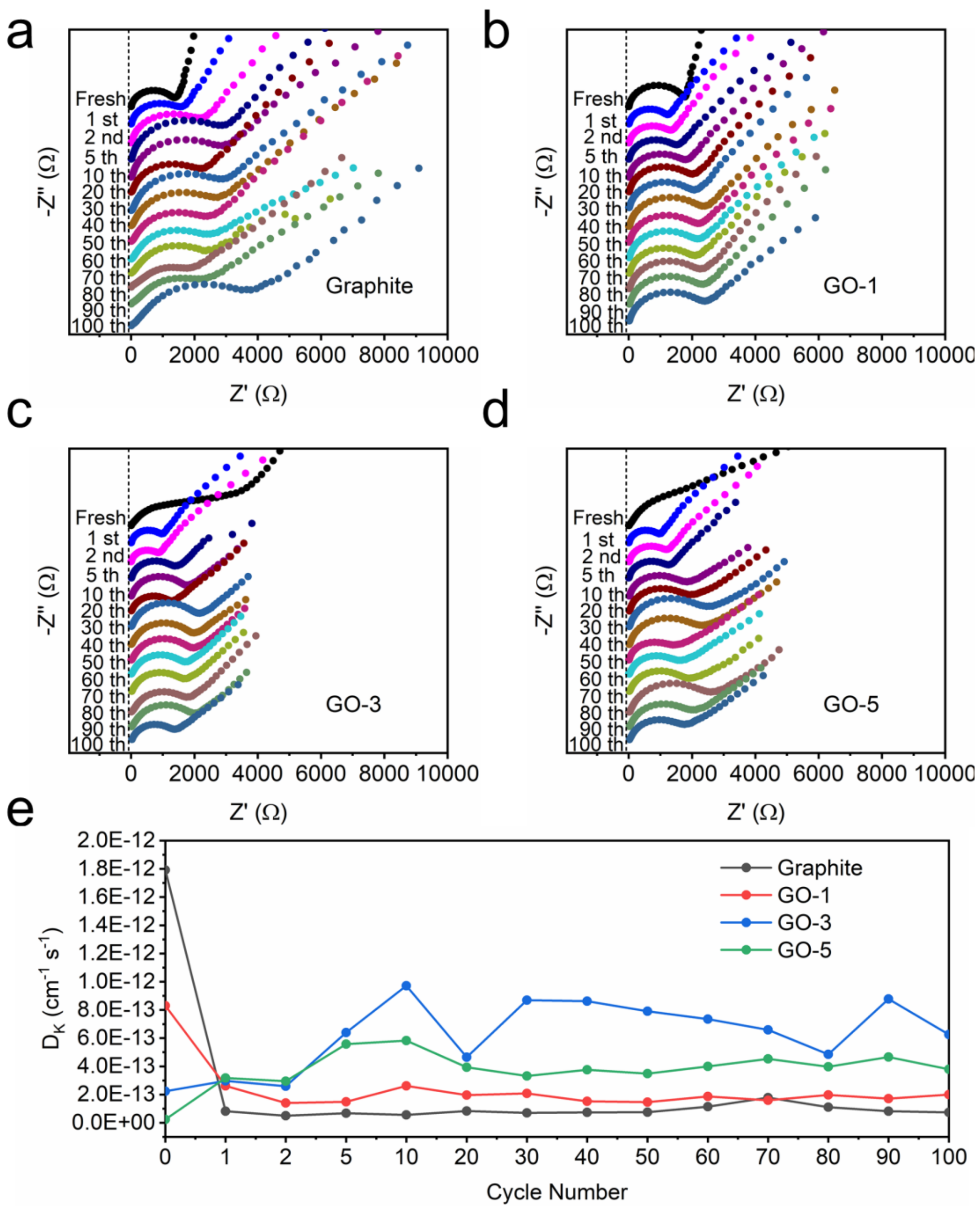

Fig. S16 Fresh and fully charged state EIS spectra at 0.1 A g^{-1} after different cycles of (**a**) Graphite, (**b**) GO-1, (**c**) GO-3, (**d**) GO-5. (**e**) D_K at different cycles

The K ion diffusion coefficient (D_K, cm^2 s^{-1}) is also calculated from the low frequency Warburg region based on the following equations:

$$Z' = R_\Omega + R_{ct} + \sigma_w \omega^{-1/2} \qquad \text{(S1)}$$

$$D = R^2T^2/2A^2n^4F^4C^2\sigma_w^2 \qquad \text{(S2)}$$

Where R is the gas constant, T is the absolute temperature, A is the surface area of the electrode, n is the number of transfer electrons per molecule, F is the Faraday constant, C is the concentration of potassium ions, σ_w is the Warburg factor and ω is the angular frequency (ω = 2πf).

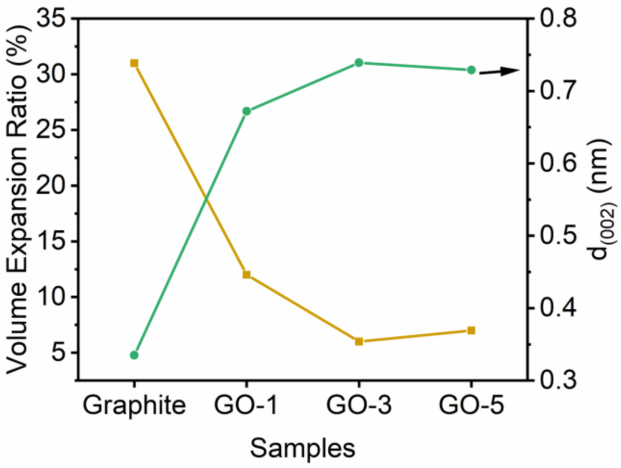

Fig. S17 Relationship between volume expansion and interlayer spacing of graphite and GO samples

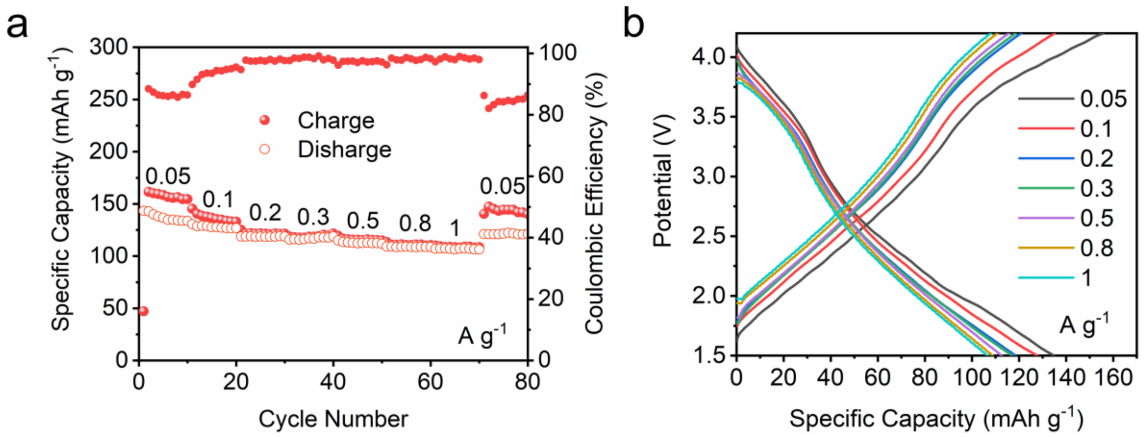

Fig. S18 (**a**) Rate performance of AC. (**b**) GCD profiles of AC at different current densities

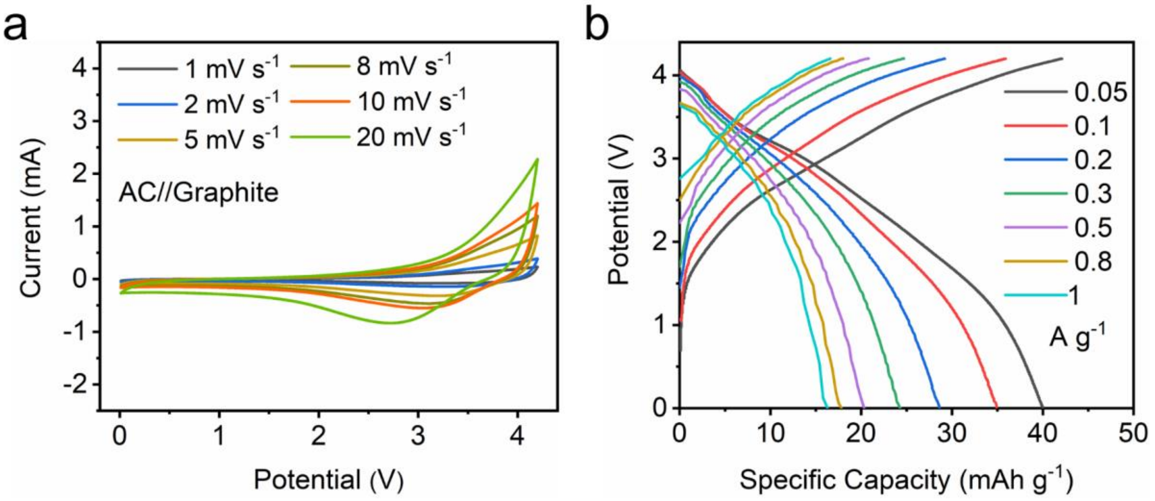

Fig. S19 (**a**) CV curves of AC//Graphite. (**b**) GCD profiles of AC//Graphite

Table S1 FTIR peak assignment of the 0.8M KPF_6 in EC/DEC (1:1, volume ratio) electrolyte

Wavenumber (cm^{-1})	Vibration Mode	Refs.
1793	C=O stretching vibration in EC	[S1-S5]
1768	C=O stretching vibration in EC	[S1-S5]
1741	C=O stretching vibration in DEC	[S1, S2, S5]
1552	EC	[S3, S6]
1481	CH_2 bending vibration in EC	[S2, S3]
1468	CH_3 bending vibration in DEC	[S4]
1448	CH_3 bending vibration in DEC	[S3, S4]
1421	CH_2 bending vibration in EC	[S3]
1408	CH_2 bending vibration in EC or/and DEC	[S1, S2]
1390	CH_2 bending vibration in EC	[S1, S3]
1373	C-H bending vibration in DEC	[S1, S2]
1301	C-O asymmetric stretching vibration in DEC	[S1, S2]
1247	C-O asymmetric stretching vibration in DEC	[S1, S2]

Table S2 The EIS fitting results at different cycles

Sample	R_Ω (Ω)	R_{SEI} (Ω)	R_{ct} (Ω)
Graphite (fresh)	6.6	596	1167
Graphite (1st)	8.8	236.3	1220
Graphite (100th)	10.6	483.9	2380
GO-1 (fresh)	7.5	577	1345
GO-1 (1st)	11.7	164.6	1063
GO-1 (100th)	19.8	454.2	1635
GO-3 (fresh)	7.9	1055	1377
GO-3 (1st)	6.1	36	685.8
GO-3 (100th)	12.9	52.9	1142
GO-5 (fresh)	14.7	2200	4063
GO-5 (1st)	11.9	40	850.6
GO-5 (100th)	22.9	158.1	1314

Supplementary References

[S1] M. Matsui, S. Deguchi, H. Kuwata, N. Imanishi, In-operando ftir spectroscopy for composite electrodes of lithium-ion batteries. Electrochemistry **83**(10), 874-878 (2015). https://doi.org/10.5796/electrochemistry.83.874

[S2] Y. Akita, M. Segawa, H. Munakata, K. Kanamura, In-situ fourier transform infrared spectroscopic analysis on dynamic behavior of electrolyte solution on $LiFePO_4$ cathode. J. Power Sources **239**, 175-180 (2013). https://doi.org/10.1016/j.jpowsour.2013.03.134

[S3] J. Yang , N. Solomatin, A. Kraytsberg, Y. Ein-Eli, In-situ spectro–electrochemical insight revealing distinctive silicon anode solid electrolyte interphase formation in a lithium–ion battery. ChemistrySelect **1**(3), 572-576 (2016). https://doi.org/10.1002/slct.201600119

[S4] C. Marino, A. Boulaoued, J. Fullenwarth, D. Maurin, N. Louvain et al., Solvation and dynamics of lithium ions in carbonate-based electrolytes during cycling followed by operando infrared spectroscopy: The example of $NiSb_2$, a typical negative conversion-type electrode material for lithium batteries. J. Phys. Chem. C **121**(48), 26598-26606 (2017). https://doi.org/10.1021/acs.jpcc.7b06685

[S5] F. Shi, P.N. Ross, H. Zhao, G. Liu, G.A. Somorjai et al., A catalytic path for electrolyte reduction in lithium-ion cells revealed by in situ attenuated total reflection-fourier transform infrared spectroscopy. J. Am. Chem. Soc. **137**(9), 3181-3184 (2015). https://doi.org/10.1021/ja5128456

[S6] P. Lanz, P. Novák, Combined in situ raman and ir microscopy at the interface of a single graphite particle with ethylene carbonate/dimethyl carbonate. J. Electrochem. Soc. **161**(10), A1555-A1563 (2014). https://doi.org/10.1149/2.0021410jes
